# Appropriate Insulin Level in Selecting Fortified Diet-Fed, Streptozotocin-Treated Rat Model of Type 2 Diabetes for Anti-Diabetic Studies

**DOI:** 10.1371/journal.pone.0170971

**Published:** 2017-01-27

**Authors:** Stanley Irobekhian Reuben Okoduwa, Ismaila A. Umar, Dorcas B. James, Hajiya M. Inuwa

**Affiliations:** 1 Department of Biochemistry, Ahmadu Bello University, Zaria, Nigeria; 2 Directorate of Research and Development, Nigerian Institute of Leather and Science Technology, Zaria, Nigeria; Max Delbruck Centrum fur Molekulare Medizin Berlin Buch, GERMANY

## Abstract

**Background:**

Pathophysiological investigation of disease in a suitable animal model is a classical approach towards development of a credible therapeutic strategy. This study examined appropriate insulin level in selecting animal model for type 2 diabetes (T2D) studies.

**Method:**

Albino Wistar rats (150-200g) were divided into two groups fed with commercially available normal-diet-feed (NDF) and water or fortified diet feed (FDF) (10g NDF per gram of margarine) with 20% fructose solution as drinking water. After 6 weeks of dietary regimen both groups were divided into 5 sub-groups and injected intraperitoneally with a graded dose of streptozotocin (STZ) (0, 25, 35, 45 & 55mg/kg bw.).

**Result:**

The result showed that the FDF-fed rats increased significantly in body weight, basal serum insulin, total cholesterol, triglycerides and blood glucose levels as compared to NDF-fed rats. Ten days post STZ induction, the groups treated with STZ (45 & 55 mg/kg) developed frank hyperglycaemia with < 46.8% serum insulin, a severe deficiency typical of diabetes type 1. The NDF_25_ and NDF_35_ groups with 75.7% and 64.4% serum insulin respectively presented relative normoglycemia, whereas the FDF_35_ (85.8% serum insulin) were notably hyperglycaemia (>300 mg/dL) throughout the 6weeks post diabetes confirmation. These FDF_35_ rats were sensitive to glibenclamide, metformin and pioglitazone in lowering hyperglycaemia, hypertriglyceridemia and hypercholesterolemia

**Conclusion:**

The hyperglycaemia stability of the FDF_35_ rats (85.5% insulin) together with their sensitivity to 3 different hypoglycaemic drugs strongly suggests their suitability as a non-genetic model of T2D. Hence the study shows that circulating serum insulin ≥ 85.8% with overt hyperglycaemia may be utilized as the benchmark in selecting rat models for T2D studies.

## Introduction

Diabetes is a lifestyle non-communicable disease of mankind considered as one of the most significant global health problems that afflict both young and old in all parts of the world irrespective of their gender [1]. The disease is a metabolic condition caused by the body’s inability to produce or make use of insulin, and it drastically decreases the quality of human life. Nigeria has the highest number of people with diabetes in Africa with 3,921,500 cases reported on a prevalence rate of 4.99% [1]. Type 2 diabetes (T2D) accounts for 95% of all cases reported [2, 3]. The causes of T2D are multi-factorial which includes both genetic and environmental elements that affect the β-cell function and insulin sensitivity [4, 5].Africa is blessed with enormous biodiversity of resources yet plagued with several diseases [6].

One of the classical approaches towards the development of credible therapeutic strategy for the possible cure of a disease is the investigation of the pathophysiology of the disease in a suitable animal model [7, 8, 9]. Despite the high prevalence of T2D and much progress made so far in basic and clinical research into diabetes, especially in our setup, where healthcare resources are limited, a final cure does not exist [10].This could possibly be due to the use of inappropriate animal model. For instance, the mode of action, *in vivo* efficacy, and side effects of anti-diabetic plants and their bioactive constituents have been comprehensively studied using inapt animal model [11, 12].

The development of vast majority of animal models of diabetes following high dose of chemical induction is primarily due to direct pancreatic beta cell destruction, resulting in insulin deficiency (which is the case for type 1 diabetes (T1D)) rather than the consequence of insulin resistance (IR) (as in the case for T2D).The use of this type of animal model would certainly not be effective in screening anti-diabetic plants/compounds intended for the management of T2D. This has been the case of many investigators [13–16].

It is therefore imperative to find a solution to the problem of T2D. To achieve this aim, a careful choice of species/strain and dietary intervention coupled with adequate control over environmental variable will be of immense significance in developing a reproducible diet-induced model of T2D [12, 17–18]. Although various diet-induced models of diabetes exist such as fructose or high-fat diet model, the pattern of initiation and development in the majority of them do not appear to be close analogues to the clinical situation in human[4,8,11]. Also, over 67% of the currently reported model of high-fat fed induced model of T2D utilized Sprague-Dawley rat and about 83% of the purportedly reported T2D model are in reality T1D because they present absolute insulin deficiency [19]. In addition, these strain/species of rats and diet compositions used apart from being very expensive are also not readily available for T2D studies especially in this part of the country [17]. Hence, there has been a continuous search by various investigators, especially in Nigeria and environs for a more readily available, cost-effective animal model that can be replicated with ease without facing reproducibility issues. This could be achieved by way of developing a new methodology or modifying the currently available procedures or a combination of both and at the same time examining their residual serum insulin status.

A prominent distinguishing feature between T1D and T2D is the residual amount of serum insulin or C-peptide level[20–22]. These biomarkers are more often not verified to ascertain the model in question during anti-diabetic screening. The majority of available literatures on diabetic screening actually make do with T1D model which involves direct destruction of pancreatic β-cells, consequently resulting in severe/absolute insulin deficiency rather than insulin resistance [13,15,16,23]. In order to remedy the problem, it has been recommended that researchers should at least provide these data (insulin/C-Peptide level) which can be used to ascertain the type and/or stage of diabetes, whether early or late phase [19].To this effect, the present study investigated these biomarkers in rat model of diabetes in a bid to ascertain a suitable model and appropriate insulin status for T2D studies.

## Materials and Methods

### Experimental Animals

Wistar Albino rats weighing 150-200g were used for the research. They were obtained from the animal house of the Department of Pharmacology, Ahmadu Bello University, Zaria-Nigeria. The rats were kept in properly ventilated cages where bedding was replaced daily, at a room temperature of about 27°C and 12 hours light/dark cycle. They were allowed to acclimatise for two weeks prior to experimentation. During this period, they were all provided with the same commercially available normal diet fed (NDF)(Grand Cereals Ltd, Nigeria) and tap water *ad libitum*. Their weights were also noted. The experimental protocol was reviewed and approved by the Animal Research Ethics Committee of Nigerian Institute of Leather and Science Technology, Zaria Nigeria (Reference Number: AREC/EA15/038) All protocol was in conformity with the institutional guidelines that are in compliance with National and International Laws and Guidelines for Care and Use of Laboratory Animals in Biomedical Research. The rules and regulations in accordance with the Ethical Committee’s directive were strictly adhered to. Efforts were made to minimize suffering. The criterion of anaesthesia was the lack of body or limb movement in response to a standardised tail clamping stimulus.

### Chemicals and Reagents

Streptozotocin (STZ) was purchased from Adooq Bioscience, LLC, USA. Glucometer strips, Mission cholesterol Meter (Acon Laboratories Ins, USA), fructose solution (Kem Light Laboratories PVT Ltd, India), Simas Margarine (PT Salim Ivomas Pratama Tbk, Indonesia), Ultrasensitive rat insulin/ rat C-peptide ELISA kits and every other reagent were of available analytical grade purchased from the appropriate manufacturing company through BioRapid Diagnostics Nigeria Limited, Abuja.

### Experimental Design and Induction of Diabetes

Two different protocols were used to induce diabetes in the rats. The rats were first allocated into two dietary groups of 40 rats namely the NDF and FDF groups.

**Protocol 1:** The rats allocated to the NDF group were initially placed on NDF and water for 6 weeks. Thereafter divided into 5 subgroups of 8 rats and given single intra-peritoneal injection (*ip*) of 0, 25, 35, 45, 55 mg/kg b.w. STZ dissolved in 0.1 ml fresh cold citrate buffer pH 4.5 into overnight fasted rats. The rats were provided 5% glucose solution as drinking water in the first 24 hrs after STZ induction.

**Protocol 2:** The other 40 rats allocated FDF group were treated with some modification of the combined methods described by Srinivasan *et al*., [24], Zhang *et al*., [12], Wilson and Islam [25]. In brief; the commercially available NDF was fortified with Simas Margarine in a ratio of 10g NDF (Crude protein 13%, fat 8%, crude fibre 15%, calcium 0.90%, available phosphorus 0.35%, methionine 0.37%, lysine 0.70%, metabolizable energy 2600kcal/kg) per gram of Margarine (fat 99.9%,Emulsifiant E471, E322, Antioxidant E304, E306, Beta Carotene C175130, Vitamin D 30,000 IU/kg).This was administered together with 20% fructose solution as drinking water to the rats *ad libitum* for 6 weeks. After which they were fasted overnight, divided into 5 subgroups of 8 rats and each rat was injected (*ip*) with a single dose of STZ (0, 25, 35, 45, 55 mg/kg b.w.) dissolved in a citrate buffer, pH 4.5.The rats were provided 5% glucose solution as drinking water in the first 24 hrs after STZ induction.

### Mean Fluid and Feed Intake

The mean food and fluid intake of all the experimental animals were taken daily and recorded.

### Confirmation of Diabetes

Confirmation was done 72 h after STZ induction, using glucose test strips and glucometer (On-Call Plus, Acon Laboratories Ins, USA). Blood samples were obtained from the tail puncture of the rats.

### Validation of Diabetes

Validation of diabetes was done 7 days after initial confirmation. Firstly, by measuring blood glucose obtained by single prick on the tail tip (allowing only one drop to come off) using glucometer in order to ensure stable hyperglycaemia. Animals with non-fasting blood glucose (NFBG) ≥ 300 mg/dL (16.7 mM) were considered diabetic and included in the study as diabetic animals [24, 26]. Secondly, oral glucose tolerance test was performed after an overnight fast.

### Oral Glucose Tolerance Test

The ability of the animals to tolerate glucose loading was examined using the oral glucose tolerance test (OGTT) performed after an overnight fast. In this test, a single dose of glucose solution (2.0 g/kg bw) was orally ingested to each animal and the levels of blood glucose obtained via tail puncture were measured at 0 (just before glucose ingestion), 30, 60, 90 and 120 min after the ingestion of glucose.

### Stability of Experimental Diabetic Model and Validation for Pharmacological Screening

The stability of the experimental animal model of diabetes was investigated for 6 weeks post validation of diabetes (i.e. 8weeks post STZ induction).Thereafter, the suitability of the model for screening purposes and pharmacological testing was established using three confirmed anti-diabetic drugs with different mechanism of actions for type 2 diabetes. The diabetic rats were treated once daily for 7 days using pioglitazone (10 mg/kg bw), metformin (500mg/kg bw) or glibenclamide (5 mg/kg bw) orally. The drugs were dissolved in 1% sodium carboxymethyl cellulose as vehicle. After the one week of oral drug administration, blood sample was withdrawn for determination of blood glucose, total cholesterol, triglyceride and serum insulin level.

### Blood Sample Collection

Blood sample was drawn from the tip of the tail and tested using glucose test strips and glucometer (On-Call Plus, Acon Laboratories Inc, USA) for blood glucose;3-in1cholesterol devise and mission cholesterol meter (Acon Laboratories Inc, USA)was used to determine the blood triglyceride and total cholesterol after overnight fast before and after the dietary regimen, and subsequently before and after STZ induction. At the end of the experimental period, animals were euthanized by halothane anaesthesia. The terminal blood collected was done after overnight fasting for the determination of Homeostatic Model Assessment (HOMA) score for insulin resistance and β-cell function (HOMA-IR and HOMA-β) which were calculated using the following formula [27]:

$$\text{HOMA}-\text{IR}=\frac{\text{Insulin}\;\left(\text{U}/\text{l}\right)\times \text{Blood glucose}\;\left(\text{mmol}/\text{l}\right)}{22.5}$$

$$HOMA-\beta =\frac{20\times \text{Insulin}\;\left(\text{U}/\text{l}\right)}{\text{Blood glucose}\;\left(\text{mmol}/\text{l}\right)}-3.5$$

Conversion factor: Insulin (1U/l = 7.174 pmol/l) and blood glucose (1 mmol/l = 18 mg/dl).

### Statistical Analyses

The results obtained were expressed as mean ± standard deviation where applicable. The data were analyzed using analysis of variance (ANOVA) and significant differences among means were determined by Duncan's Multiple Range Test at p< 0.05 using SPSS software version 20 for windows.

## Result

### Effects of Streptozotocin Treatment on Fluid and Feed Intake of Experimental Rats

**Fig 1:**
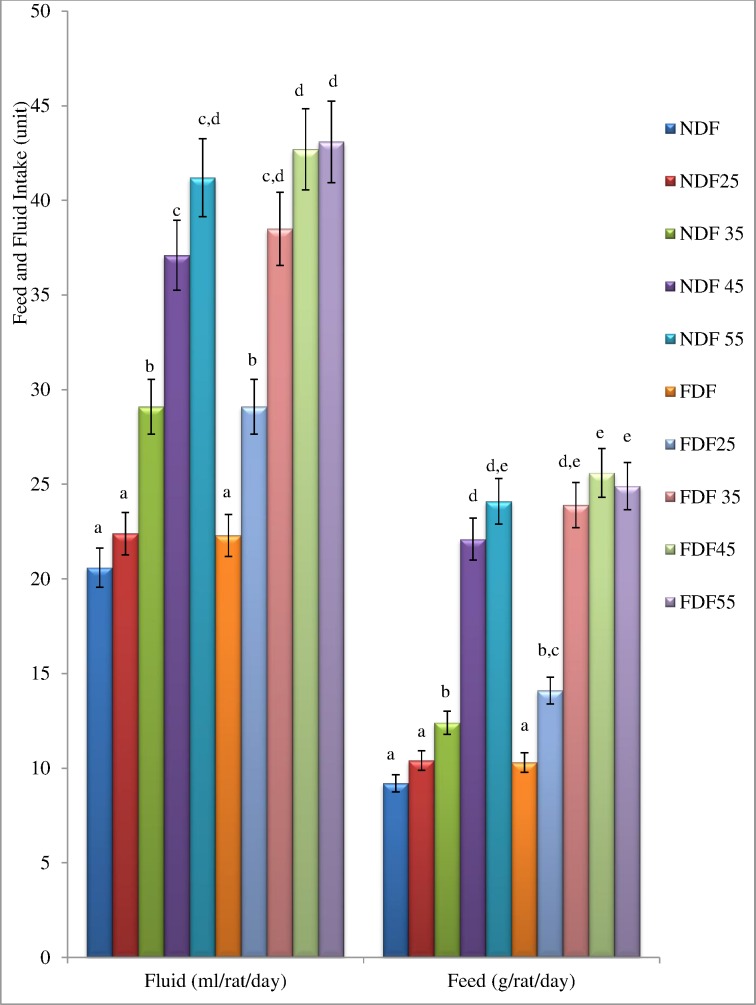
Mean Fluid Intake and Feed Intake of each Animal in the Groups per Day during the Experimental Period. Data are presented as mean ± SD of 6–8 animals per group. Bars with different superscript indicate significant difference (P<0.05). **NDF:** Group fed with Normal Diet Feed + water and treated with normal saline as control **NDF**_**25**_**:** Group fed with Normal Diet Feed + Water and treated with 25mg/kg bw, STZ. **NDF**_**35**_**:** Group fed with Normal Diet Feed + Water and treated with 35mg/kg bw, STZ. **NDF**_**45**_**:** Group fed with Normal Diet Feed + Water and treated with 45mg/kg bw, STZ **NDF**_**55**_**:** Group fed with Normal Diet Feed + Water and treated with 55mg/kg bw, STZ. **FDF:** Group fed with Fortified Diet Feed + 20% fructose and treated with Normal saline **FDF**_**25**_**:** Group fed with Fortified Diet Feed + 20% fructose and treated with 25mg/kg bw STZ **FDF**_**35**_**:** Group fed with Fortified Diet Feed + 20% fructose and treated with 35mg/kg bw STZ **FDF**_**45**_**:** Group fed with Fortified Diet Feed + 20% fructose and treated with 45mg/kg bw STZ **FDF**_**55**_**:** Group fed with Fortified Diet Feed + 20% fructose and treated with 55mg/kg bw STZ.

The mean fluid and feed intake per animal per day throughout the experimental duration is presented in Fig 1.The fluid and feed intake of the STZ treated rats were significantly higher (P<0.05) compared to NDF and FDF groups. This effect was observed in the STZ treated groups with frank hyperglycaemia.

### Effect of Dietary Regimen and Streptozotocin Treatment on Body Weight of Rats

The effect of 6 weeks dietary regimen on mean body weight of animals in each group is presented in Table 1. It was observed that the group administered combined FDF and 20% fructose solution had significantly increased (*p*<0.05) body weight as compared to the NDF-fed group. The percentage change in body weight was approximately2 folds in the FDF-fed group as against the NDF-fed group indicating a state of obesity in the FDF-fed rats. Ten days post STZ induction it was observed that the groups treated with fairly high STZ dose (45 and 55 mg/kg bw.*i*.*p*.) had drastically reduced body weight; the percentage change in their body weight was between 4.57–11.97%. Whereas those administered low dose of STZ (25 mg/kg bw) had smaller in weight gained when compared to the control groups treated with vehicle alone (NDF and FDF). There was no significant difference (p >0.05) in the relative decrease in weight between the FDF-fed and NDF-fed groups treated with the same STZ dose of 35 mg/kg bw of STZ (Table 1).

**Table 1:** Percentage Change in Body Weight of Rats after Dietary Regimen and STZ Induction.

Group	Body Weight (g) (Day 0)	Percentage Change in Body Weight (%)	
		Day 42 of Dietary Regimen	Day 52 (Day 10 Post STZ Induction
NDF	1 70.33 ± 12.30 ^a^	14.97 ± 3.09 ^a^	2.59 ± 0.44 ^f^
NDF_25_	168.67 ± 13.94 ^a^	15.07 ± 3.00 ^a^	1.80 ± 0.16 ^e,f^
NDF_35_	173.62 ± 19.64 ^a^	14.16 ± 6.20 ^a^	-1.23 ± 1.05 ^d^
NDF_45_	167.83 ± 7.41 ^a^	15.93 ± 3.28 ^a^	-4.69 ± 0.16 ^c^
NDF_55_	172.27 ± 20.87 ^a^	13.12 ± 1.37 ^a^	-11.97 ± 1.82 ^a^
FDF	173.38 ± 16.29 ^a^	31.09 ± 4.42 ^b^	2.61 ± 1.13 ^f^
FDF_25_	163.92 ± 8.65 ^a^	36.68 ± 3.25 ^b^	1.14 ± 0.52 ^e^
FDF_35_	162.87 ± 8.29 ^a^	34.54 ± 6.25 ^b^	-1.16 ± 1.31 ^d^
FDF_45_	169.97 ± 10.80 ^a^	33.31 ± 4.74 ^b^	-4.57 ± 1.14 ^c^
FDF_55_	171.40 ± 9.93 ^a^	33.09 ± 5.91 ^b^	-9.08 ± 1.01 ^b^

Data are presented as mean ± SD of 6–8 animals per group. Values with different superscript down the column indicate significant difference (P<0.05). **NDF:** Group fed with Normal Diet Feed + water and treated with normal saline as control; **NDF**_**25**_**:** Group fed with Normal Diet Feed + Water and treated with 25mg/kg bw, STZ.; **NDF**_**35**_**:** Group fed with Normal Diet Feed + Water and treated with 35mg/kg bw, STZ.; **NDF**_**45**_**:** Group fed with Normal Diet Feed + Water and treated with 45mg/kg bw, STZ; **NDF**_**55**_**:** Group fed with Normal Diet Feed + Water and treated with 55mg/kg bw, STZ.; **FDF:** Group fed with Fortified Diet Feed + 20% fructose and treated with Normal saline; **FDF**_**25**_**:** Group fed with Fortified Diet Feed + 20% fructose and treated with 25mg/kg bw STZ; **FDF**_**35**_**:** Group fed with Fortified Diet Feed + 20% fructose and treated with 35mg/kg bw STZ; **FDF**_**45**_**:** Group fed with Fortified Diet Feed + 20% fructose and treated with 45mg/kg bw STZ; **FDF**_**55**_**:** Group fed with Fortified Diet Feed + 20% fructose and treated with 55mg/kg bw STZ.

### Evaluation of Non-Fasting Blood Glucose Levels during Post Dietary Treatment and STZ Induction in Rats

Table 2 illustrates that after 6 weeks of dietary regimen the fasting blood glucose of the FDF-fed group had increased significantly (p <0.05) as compared to the NDF-fed group. At day-3 post-STZ induction, there was significant increase in non-fasting blood glucose in the entire STZ treated groups as compared to the vehicle treated control groups. Ten days post-STZ induction, it was noticed that the fasting blood glucose level in the NDF_25_, NDF_35_, NDF_45_ and FDF_25_ had significantly reduced (p<0.05) when compared to values obtained on day-3 post-STZ induction indicating a form of reversal effect. However, the NDF_55_, FDF_35_, FDF_45_ and FDF_55_ groups had increased non-fasting blood glucose levels when compared to the values obtained on day-3 post-STZ induction.

**Table 2:** Non-Fasting Blood Glucose (mg/dL) after Dietary Regimen and STZ Induction.

	Dietary Regimen			
	Day 0	Day 42		
Group		Post STZ Induction		
		Day 0	Day 3	Day 10
NDF	85.63 ± 7.05 ^a^	84.87 ± 8.18 ^a^	84.61 ± 7.78 ^a^	85.10 ± 7.76 ^a^
NDF_25_	88.67 ± 5.33 ^a^	87.80 ± 3.88 ^a^	94.80 ± 3.47 ^a^	88.05 ± 1.80 ^a,b^
NDF_35_	85.90 ± 7.10 ^a^	86.23 ± 4.74 ^a^	120.92 ± 4.93 ^c^	101.80 ± 5.61 ^b,c^
NDF_45_	87.73 ± 3.36 ^a^	89.40 ± 3.36 ^a^	191.45 ± 10.85 ^d^	183.62 ± 10.56 ^d^
NDF_55_	88.17 ± 3.40 ^a^	87.40 ± 3.58 ^a^	340.97 ± 22.49 ^f^	356.18 ± 22.16 ^f^
FDF	84.82 ± 4.83 ^a^	102.07 ± 3.50 ^b^	102.25 ± 3.13 ^a,b^	103.47 ± 5.47 ^b,c^
FDF_25_	85.93 ± 6.60 ^a^	100.68 ± 4.58 ^b^	119.03 ± 3.91 ^b,c^	114.93 ± 5.71 ^c^
FDF_35_	85.83 ± 4.95 ^a^	100.70 ± 4.93 ^b^	303.48 ± 25.93 ^e^	309.42 ± 19.56 ^e^
FDF_45_	88.13 ± 3.82 ^a^	100.38 ± 2.37 ^b^	344.65 ± 22.56 ^f^	371.73 ± 17.79 ^f^
FDF_55_	81.52 ± 4.97 ^a^	98.70 ± 2.37 ^b^	487.57 ± 18.59 ^g^	507.08 ± 14.50 ^g^

Data are presented as mean ± SD of 6–8 animals per group. Values with different superscript down the column indicate significant difference (P<0.05). **NDF:** Group fed with Normal Diet Feed + water and treated with normal saline as control; **NDF**_**25**_**:** Group fed with Normal Diet Feed + Water and treated with 25mg/kg bw, STZ.; **NDF**_**35**_**:** Group fed with Normal Diet Feed + Water and treated with 35mg/kg bw, STZ.; **NDF**_**45**_**:** Group fed with Normal Diet Feed + Water and treated with 45mg/kg bw, STZ; **NDF**_**55**_**:** Group fed with Normal Diet Feed + Water and treated with 55mg/kg bw, STZ.; **FDF:** Group fed with Fortified Diet Feed + 20% fructose and treated with Normal saline; **FDF**_**25**_**:** Group fed with Fortified Diet Feed + 20% fructose and treated with 25mg/kg bw STZ; **FDF**_**35**_**:** Group fed with Fortified Diet Feed + 20% fructose and treated with 35mg/kg bw STZ; **FDF**_**45**_**:** Group fed with Fortified Diet Feed + 20% fructose and treated with 45mg/kg bw STZ; **FDF**_**55**_**:** Group fed with Fortified Diet Feed + 20% fructose and treated with 55mg/kg bw STZ.

### Serum Total Cholesterol and Triglyceride Levels in the Experimental Rats

Table 3 shows the effect of the dietary regimen and STZ induction on total cholesterol (TC) and triglyceride (TG) levels in the experimental rats. It was observed that the serum TC and TG levels had increased significantly (p > 0.05) in the FDF groups after the 6 weeks dietary regimen. Ten days post STZ induction, the serum and TG levels in all the STZ treated groups had increased significantly (p >0.05) when compared to the control groups (FDF and NDF). The increased in serum TC and TG levels were more severe in the FDF_55_, FDF_45_, FDF_35_ and NDF_55_groups.

**Table 3:** Serum Total Cholesterol and Triglyceride in Experimental Rats.

Group	Day 0 of Dietary Regimen		Day 42 of Dietary Regimen (Day 0, STZ Induction)		Day 10, Post STZ Induction	
	Serum TC	Serum TG	Serum TC	Serum TG	Serum TC	Serum TG
NDF	78.7 ± 4.56 ^a^	68.7 ± 5.91 ^a^	81.9 ± 9.84 ^a^	70.5 ± 5.92 ^a^	83.6 ± 6.82 ^a^	69.4 ± 6.80 ^a^
NDF_25_	85.6 ± 7.45 ^a^	75.4 ± 8.84 ^a^	88.7 ± 7.86 ^a^	73.9 ± 6.84 ^a^	109.4 ± 6.93 ^b^	87.5 ± 5.92 ^b^
NDF_35_	80.2 ± 4.38 ^a^	81.2 ± 8.96 ^a^	83.5 ± 7.55 ^a^	80.4 ± 4.86 ^a^	102.7 ± 8.95 ^b^	94.2 ± 7.85 ^b^
NDF_45_	74.3 ± 7.94 ^a^	74.6 ± 7.95 ^a^	79.6 ± 8.38 ^a^	77.3 ± 5.92 ^a^	135.4 ± 7.97 ^d^	276.3 ± 7.04 ^d^
NDF_55_	83.1 ± 6.38 ^a^	76.8 ± 8.87 ^a^	87.2 ± 5.95 ^a^	74.5 ± 4.85 ^a^	141.9 ± 8.89 ^d^	423.7 ± 8.58 ^f^
FDF	77.4 ± 7.94 ^a^	69.7 ± 6.00 ^a^	103.4 ± 6.93 ^b^	119.5 ± 8.42 ^b^	102.2 ± 7.92 ^b^	224.6 ± 6.84 ^c^
FDF_25_	82.7 ± 4.58 ^a^	74.8 ± 8.82 ^a^	114.6 ± 7.39 ^b^	133.4 ± 6.82 ^b^	117.3 ± 8.72 ^c^	338.3 ± 7.06 ^e^
FDF_35_	79.5 ± 5.72 ^a^	80.2 ± 7.90 ^a^	115.7 ± 8.55 ^b^	129.2 ± 9.80 ^b^	174.1 ± 9.57 ^e^	420.2 ± 9.04 ^f^
FDF_45_	86.5 ± 6.83 ^a^	67.3 ± 9.37 ^a^	103.8 ± 8.07 ^b^	132.5 ± 4.96 ^b^	185.3 ± 5.85 ^e^	431.5 ± 6.85 ^f^
FDF_55_	81.2 ± 8.29 ^a^	72.6 ± 8.35 ^a^	102.5 ± 9.35 ^b^	127.6 ± 7.82 ^b^	173.4 ± 8.20 ^e^	452.0 ± 6.92 ^g^

Data are presented as mean ± SD of 6–8 animals per group. Values with different superscript down the column indicate significant difference (P<0.05). **TC:** Total Cholesterol; **TG:** Triglyceride; **NDF:** Group fed with Normal Diet Feed + water and treated with normal saline as control; **NDF**_**25**_**:** Group fed with Normal Diet Feed + Water and treated with 25mg/kg bw, STZ.; **NDF**_**35**_**:** Group fed with Normal Diet Feed + Water and treated with 35mg/kg bw, STZ.; **NDF**_**45**_**:** Group fed with Normal Diet Feed + Water and treated with 45mg/kg bw, STZ; **NDF**_**55**_**:** Group fed with Normal Diet Feed + Water and treated with 55mg/kg bw, STZ.; **FDF:** Group fed with Fortified Diet Feed + 20% fructose and treated with Normal saline; **FDF**_**25**_**:** Group fed with Fortified Diet Feed + 20% fructose and treated with 25mg/kg bw STZ; **FDF**_**35**_**:** Group fed with Fortified Diet Feed + 20% fructose and treated with 35mg/kg bw STZ; **FDF**_**45**_**:** Group fed with Fortified Diet Feed + 20% fructose and treated with 45mg/kg bw STZ; **FDF**_**55**_**:** Group fed with Fortified Diet Feed + 20% fructose and treated with 55mg/kg bw STZ.

### Oral Glucose Tolerance Test in FDF-fed, NDF-fed and STZ-Treated Diabetic Rats

**Fig 2:**
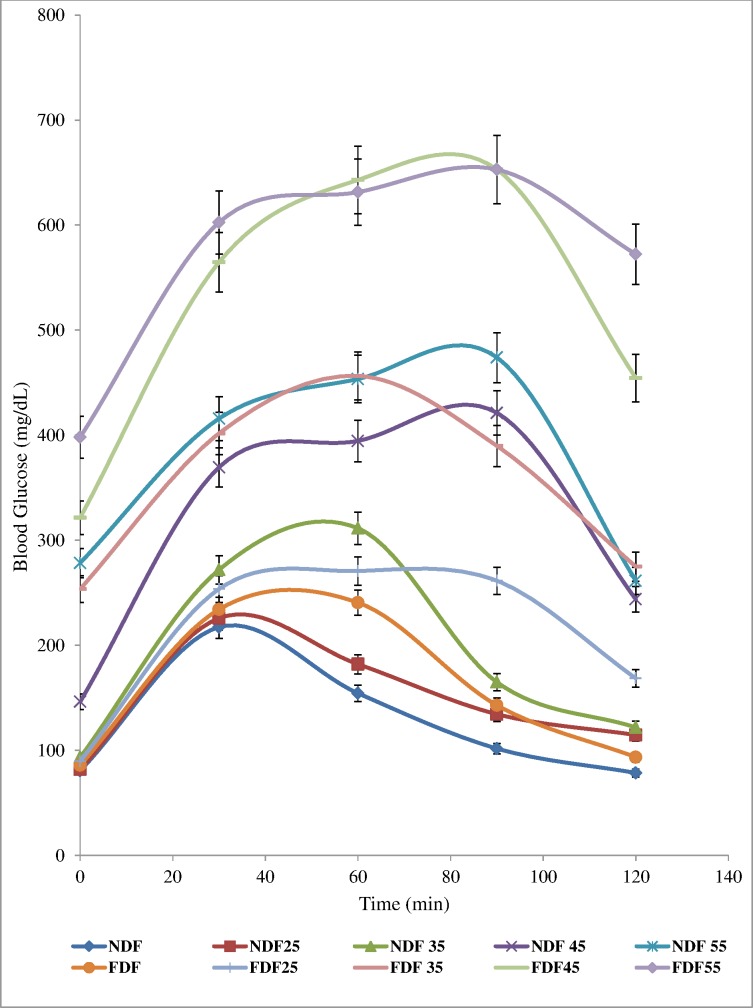
Oral Glucose Tolerance Test. Data are presented as mean ± SD of 6–8 animals per group. **NDF:** Group fed with Normal Diet Feed + water and treated with normal saline as control **NDF**_**25**_**:** Group fed with Normal Diet Feed + Water and treated with 25mg/kg bw, STZ. **NDF**_**35**_**:** Group fed with Normal Diet Feed + Water and treated with 35mg/kg bw, STZ. **NDF**_**45**_**:** Group fed with Normal Diet Feed + Water and treated with 45mg/kg bw, STZ **NDF**_**55**_**:** Group fed with Normal Diet Feed + Water and treated with 55mg/kg bw, STZ. **FDF:** Group fed with Fortified Diet Feed + 20% fructose and treated with Normal saline **FDF**_**25**_**:** Group fed with Fortified Diet Feed + 20% fructose and treated with 25mg/kg bw STZ **FDF**_**35**_**:** Group fed with Fortified Diet Feed + 20% fructose and treated with 35mg/kg bw STZ **FDF**_**45**_**:** Group fed with Fortified Diet Feed + 20% fructose and treated with 45mg/kg bw STZ **FDF**_**55**_**:** Group fed with Fortified Diet Feed + 20% fructose and treated with 55mg/kg bw STZ.

The data for OGTT showed glycaemia peak after 90 min before falling sharply in the groups treated with 45 and 55 mg/kg bw STZ. The FDF_35_glycaemia levels were maintained elevated >300 mg/dL nearly althrough the duration of 120 min glucose tolerance test with a peak at 60 min. The control groups (NDF and FDF) and the 25mg/kg bw STZ treated groups (NDF_25_ and FDF_25_) revealed a glycaemia peak at 30 min which fall sharply to almost baseline after 2 hours (Fig 2)

### Serum Insulin and C-Peptide Level in FDF-fed, NDF-fed and STZ-Treated Diabetic Rats

**Fig 3:**
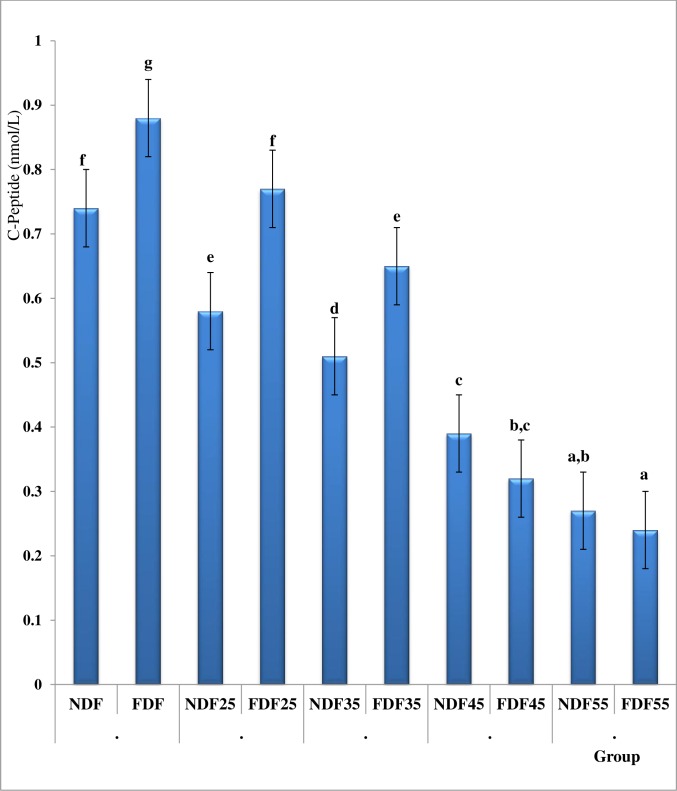
Fasting Serum C-Peptide Levels in Different Animal Groups. Data are presented as mean ± SD of 6–8 animals per group. Bars with different superscript indicate significant difference (P<0.05). Normal serum C-Peptide is 0.17–0.90 nmol/L. **NDF:** Group fed with Normal Diet Feed + water and treated with normal saline as control **NDF**_**25**_**:** Group fed with Normal Diet Feed + Water and treated with 25mg/kg bw, STZ. **NDF**_**35**_**:** Group fed with Normal Diet Feed + Water and treated with 35mg/kg bw, STZ. **NDF**_**45**_**:** Group fed with Normal Diet Feed + Water and treated with 45mg/kg bw, STZ **NDF**_**55**_**:** Group fed with Normal Diet Feed + Water and treated with 55mg/kg bw, STZ. **FDF:** Group fed with Fortified Diet Feed + 20% fructose and treated with Normal saline **FDF**_**25**_**:** Group fed with Fortified Diet Feed + 20% fructose and treated with 25mg/kg bw STZ **FDF**_**35**_**:** Group fed with Fortified Diet Feed + 20% fructose and treated with 35mg/kg bw STZ **FDF**_**45**_**:** Group fed with Fortified Diet Feed + 20% fructose and treated with 45mg/kg bw STZ **FDF**_**55**_**:** Group fed with Fortified Diet Feed + 20% fructose and treated with 55mg/kg bw STZ.

The Fasting serum insulin and C-peptide in the FDF-fed groups were significantly higher when compared to the NDF-fed groups. The injection of STZ (45 and 55 mg/kg b.w. *i*.*p*.) to the rats after 6 weeks of dietary regimen resulted in significant decrease in insulin and C-peptide both in the NDF-fed and FDF-fed groups in an equivalent amount. However, the Fasting serum C-peptide and serum insulin levels in the FDF_25_and FDF_35_ groups where comparatively high when compared to the NDF_25_ and NDF_35_ groups but lower when compared to the NDF and FDF groups (Fig 3 and Table 4).

**Table 4:** Effect of STZ Dose on Percentage Change in Serum Insulin, C-Peptide and Blood Glucose levels in all the groups with respect to the NDF control group.

Group	Percentage Change in Serum C-Peptide (%)	Circulating Serum Insulin (mU/L)	Percentage of Circulating Serum Insulin (%)	Blood Glucose (mg/dL)
NDF	-	13.47 ± 1.31	-	85.10 ± 7.76
NDF_25_	-21.62	10.20 ± 1.20	75.72	88.05 ± 1.80
NDF_35_	-31.08	8.67 ± 1.18	64.37	101.80 ± 5.61
NDF_45_	-47.3	6.30 ± 1.24	46.77	183.62 ± 10.56
**NDF**_**55**_	-63.51	4.08 ± 1.29	30.29	356.18 ± 22.16
FDF	18.92	15.82 ± 1.27	117.45	103.47 ± 5.47
FDF_25_	4.05	14.74 ± 0.35	109.43	114.93 ± 5.71
**FDF**_**35**_	-12.16	11.55 ± 1.28	85.75	309.42 ± 19.56
FDF_45_	-56.76	5.10 ± 1.24	37.86	371.73 ± 17.79
FDF_55_	-67.57	3.30 ± 1.16	24.5	507.08 ± 14.50

Data are presented as mean ± SD of 6–8 animals per group. **NDF:** Group fed with Normal Diet Feed + water and treated with normal saline as control. **NDF**_**25**_**:** Group fed with Normal Diet Feed + Water and treated with 25mg/kg bw, STZ.; **NDF**_**35**_**:** Group fed with Normal Diet Feed + Water and treated with 35mg/kg bw, STZ.; **NDF**_**45**_**:** Group fed with Normal Diet Feed + Water and treated with 45mg/kg bw, STZ; **NDF**_**55**_**:** Group fed with Normal Diet Feed + Water and treated with 55mg/kg bw, STZ.; **FDF:** Group fed with Fortified Diet Feed + 20% fructose and treated with Normal saline; **FDF**_**25**_**:** Group fed with Fortified Diet Feed + 20% fructose and treated with 25mg/kg bw STZ; **FDF**_**35**_**:** Group fed with Fortified Diet Feed + 20% fructose and treated with 35mg/kg bw STZ; **FDF**_**45**_**:** Group fed with Fortified Diet Feed + 20% fructose and treated with 45mg/kg bw STZ; **FDF**_**55**_**:** Group fed with Fortified Diet Feed + 20% fructose and treated with 55mg/kg bw STZ.

### Percentage Change in Serum C-Peptide and Circulating Serum Insulin Levels

The percentage change in fasting serum C-Peptide and circulating serum insulin levels are presented in Fig 3 and Table 4. It was observed that the FDF_45_, FDF_55_ and NDF_55_ presented frank hyperglycaemia with significant (p <0.05) decrease in circulating serum insulin and C-peptides, a severe decrease which is classical of type 1 diabetes. Interestingly, the NDF_35_ and NDF_25_ with 64.37% and 75.72% available circulating serum insulin respectively were relatively normoglycemia whereas the FDF_35_ with 85.75% circulating serum insulin had hyperglycaemia, which is distinctive feature seen among type 2 diabetic subjects.

### Assessment of Insulin Sensitivity, HOMA-IR, HOMA-β Scores in Different Animal Groups

In Table 5 the calculated insulin sensitivity index, HOMA-IR and HOMA-β scores are shown for all the groups of animal. It was observed that pancreatic β-cell function (HOMA-β score) decreases with increased in STZ dosage. The HOMA-IR score was observed to be highest in the FDF_35_ group. The least value of insulin sensitivity was also noted shown by the FDF_35_ group.

**Table 5:** Evaluation of the Beta-Cell Function and Insulin Sensitivity/Resistance of the Rats.

S/N	Group	HOMA-IR	HOMA-B	Insulin Sensitivity
1	NDF	2.83 ± 0.33 ^a,b^	53.98 ± 8.38 ^e^	1.33 ± 0.09 ^b,c^
2	NDF_25_	2.22 ± 0.26 ^a^	38.23 ± 5.01 ^d^	1.44 ± 0.07 ^b,c^
3	NDF_35_	2.18 ± 0.27 ^a^	27.31 ± 5.18 ^c^	1.43 ± 0.10 ^b,c^
4	NDF_45_	2.86 ± 0.65 ^a,b^	8.85 ± 2.22 ^b^	1.27 ± 0.15 ^b,c^
5	NDF_55_	3.59 ± 1.14 ^b,c^	0.64 ± 1.36 ^a^	1.40 ± 0.45 ^b,c^
6	FDF	4.15 ± 0.39 ^c^	53.29 ± 6.77 ^e^	1.09 ± 0.04 ^a,b^
7	FDF_25_	3.99 ± 0.35 ^c^	40.58 ± 3.79 ^d^	1.08 ± 0.05 ^a,b^
8	FDF_35_	8.85 ± 1.36 ^d^	29.94 ± 1.22 ^c^	0.77 ± 0.04 ^a^
9	FDF_45_	4.66 ± 1.01^c^	1.48 ± 1.37 ^a^	1.11 ± 0.16 ^a,b^
10	FDF_55_	4.16 ± 1.53 ^c^	1.17 ± 0.78 ^a^	1.67 ± 0.92 ^c^

Data are presented as mean ± SD of 6–8 animals per group. Values with different superscript down the column indicate significant difference (p<0.05). **NDF:** Group fed with Normal Diet Feed + water and treated with normal saline as control; **NDF**_**25**_**:** Group fed with Normal Diet Feed + Water and treated with 25mg/kg bw, STZ.; **NDF**_**35**_**:** Group fed with Normal Diet Feed + Water and treated with 35mg/kg bw, STZ.; **NDF**_**45**_**:** Group fed with Normal Diet Feed + Water and treated with 45mg/kg bw, STZ; **NDF**_**55**_**:** Group fed with Normal Diet Feed + Water and treated with 55mg/kg bw, STZ.; **FDF:** Group fed with Fortified Diet Feed + 20% fructose and treated with Normal saline; **FDF**_**25**_**:** Group fed with Fortified Diet Feed + 20% fructose and treated with 25mg/kg bw STZ; **FDF**_**35**_**:** Group fed with Fortified Diet Feed + 20% fructose and treated with 35mg/kg bw STZ; **FDF**_**45**_**:** Group fed with Fortified Diet Feed + 20% fructose and treated with 45mg/kg bw STZ; **FDF**_**55**_**:** Group fed with Fortified Diet Feed + 20% fructose and treated with 55mg/kg bw STZ.

### Stability and Validation of Experimental Animal Model for Pharmacological Screening

**Fig 4:**
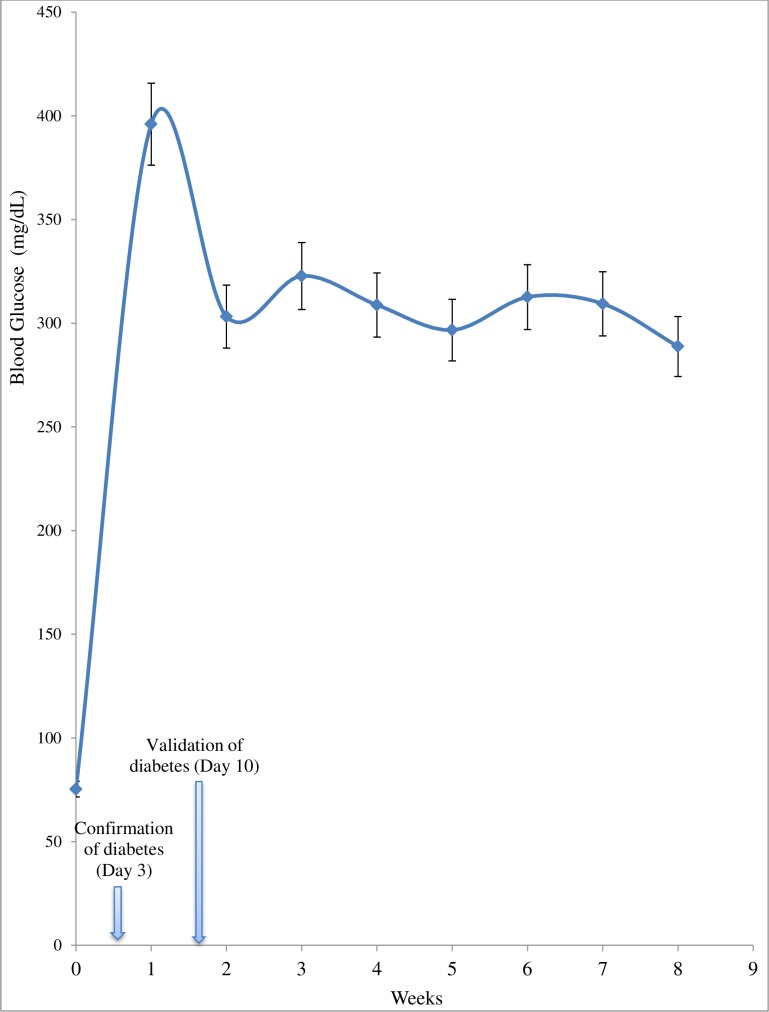
Non-Fasting Blood Glucose Curve in FDF_35_ Rat Group from Post STZ Induction to the End of the Experiment. Data are presented as mean ± SD of 6 animals.

**Fig 5:**
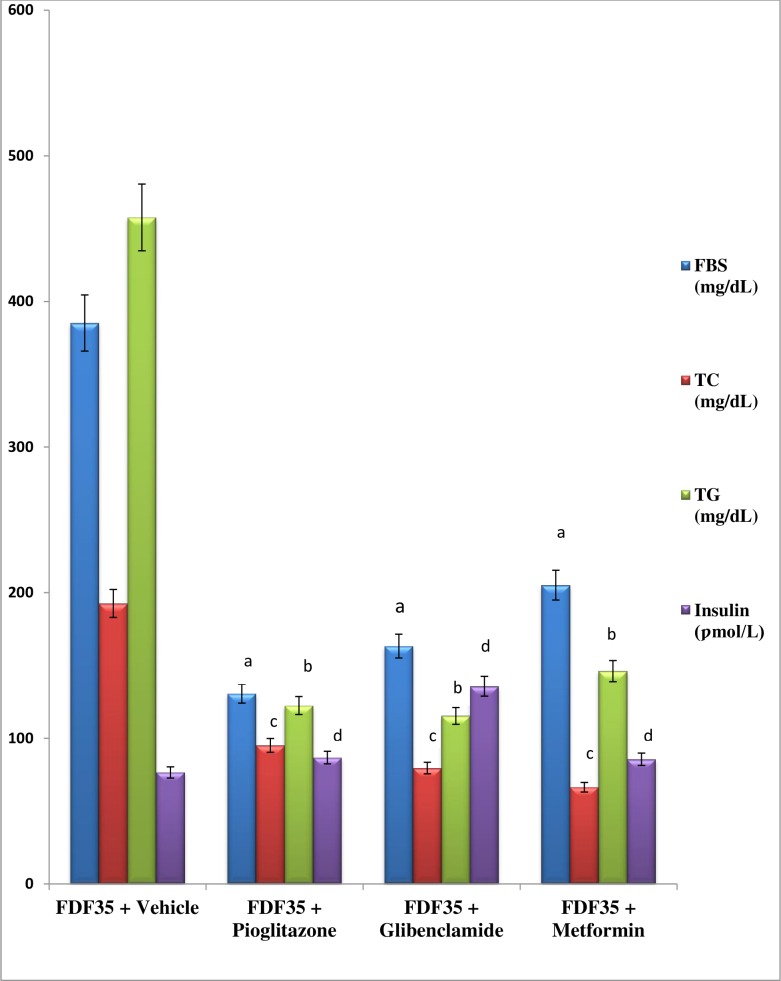
Effect of Pioglitazone, Glibenclamide and Metformin on Blood Glucose, Total Cholesterol and Triglyceride in FDF-Fed, STZ Treated Rat Model of Type 2 Diabetes. Data are presented as mean ± SD of 6 animals per group. **TC:** total cholesterol, **TG:** triglyceride, **FBS:** fasting blood sugar. **FDF**_**35**_**:** Group fed with Normal Diet Feed + Water and treated with 35mg/kg bw, STZ.“a, b, c, d”*P* ≤ 0.05versus“FDF_35_ + Vehicle”. Conversion factor: Insulin (1U/l = 7.174 pmol/l).

From the data shown in Fig 4 for FDF_35_rats group, it was observed that the basal hyperglycaemia was stable around 300 mg/dL from week 2 althrough the 6 weeks post validation duration of the experiment. The validation and suitability of the FDF_35_ rat model was established using oral administration of three confirmed type 2 diabetes anti-hyperglycaemic drugs. With metformin (500 mg/kg bw) once daily for one week, there was significantly (p<0.05) reduction in blood glucose (46.7%), TC (65.5%) and TG (68.1%) when compared to vehicle-treated diabetic rats. There was no significant (p>0.05) alteration in the insulin level and compared to the control group. Similarly, the glibenclamide (10 mg/kg bw) resulted in significant reduction in blood glucose (57.6%), TC (58.7%) and TG (74.8%). The circulating insulin levels were significantly (p<0.05) increased. The pioglitazone was observed to have also significantly reduced the blood glucose by 66.1%. The TC and TG were increased significantly (p<0.05) when compared to the vehicle treated diabetic rats (Fig 5).

## Discussion

The study was carried out with the aim to suggest and provide an easy to reproduce suitable non-genetic rat model of T2D for screening of anti-diabetic plants and secondly to ascertain the appropriate insulin level of the model. A suitable experimental model that closely mimics the pathogenesis and clinical features of T2D must adequately reflect the two prominent characteristics of human T2D which are insulin resistance and partial pancreatic beta cell dysfunction [4,12,17]. Moreover, the model must be readily available, relatively cheap and easy to develop.

In this study, Albino Wistar rats were used as the experimental animal model. The reason is that they are predominantly available and easy to breed in this part of the country unlike the Sprague-Dawley rats used by previous investigators [14,24,25,28–29]. Different strain and background species of rats have been reported to affect the susceptibilities of replicating a specific model in different geographical terrain [18,30–32] hence the need for the model presented in this study. More also, since Sprague-Dawley rats are more sensitive towards T2D than Wistar rats [33], they are less representative of the human population, where sensitivity towards T2D varies greatly from one individual to another. The Albino Wistar rats are by their varied sensitivity towards T2D, closer to the human population [33,34].

The significantly increased (*P*<0.05) food intake (polyphagia) and excessive fluid intake (polydipsia) that was observed in the STZ treated rats in this study are characteristics symptoms of diabetes mellitus. These features are usually accomplished with reduction in body weight which was noticed also in this study in the STZ treated rats. Increased water and food consumption are direct result of the accumulation of glucose in the blood and are usually dependent on the energy expenditure, urinary excretion and catabolic processes [2, 15, 34].These findings corroborate those from previous investigations [25,29,33].The observed increase in body weight among the FDF-fed groups at 6 weeks post-dietary regimen could be the result of consumption of diet rich in high fat (margarine) and fructose. The onset of T2D is strongly associated with weight gain, insulin resistance and pancreatic β-cell dysfunction. Lack of β-cells compensation results in impaired glucose intolerance [30]. This could be due to the reduced glucose tolerance observed during the OGTT in the FDF_35_ groups. Dietary fructose stimulates less insulin secretion but enhances food intake [35]. Fructose metabolism cannot be controlled by insulin or leptin, which are key factors for the regulation of fat synthesis and energy intake hence, obesity will likely be the consequence [36,37]. Obesity induced by environmental manipulation rather than gene, is thought to model the human situation more than genetic model. After 6 weeks of dietary regimen, the FDF-fed rats had significantly increased (p<0.05) bodyweight, as compared to the counterpart group of rats fed on NDF as observed in the present study. The consumption of high-fat diet such as the FDF described in the present study leads to obesity because it facilitates the development of a positive energy balance. This observation corroborates the report presented by some previous investigators [17, 30,32,38].

Previously, it was reported that 10% fructose administration *ad libitum* results in insulin resistance [25]. However, during our preliminary investigation in the present study, it was observed that providing 20% fructose alone as drinking water to Albino Wistar rats for 2 weeks could not induce insulin resistance. But when 17.1% fat enriched diet (FDF) was combined with the 20% fructose solution provided as drinking water, a state of insulin resistance was observed after 6 weeks. This observation is in agreement with the findings of Zarfeshani *et al*., [8] in which Sprague-Dawley rat fed 21% fructose solution for a period of 10 weeks could not develop signs of diabetes type 2 [8]. In another study, Stark *et al*., [39] reported that feeding rats with high fructose (53% w/v) and sucrose (10%w/v) diet was not able to assert hyperglycaemia, hyperinsulinemia, hypertriglyceridemia and hypercholesterolemia so unable to develop diabetes type 2 model among rats. These observations and ours were contrary to that of Wilson and Islam [25] who reported that a 10% fructose administration *ad libitum* resulted in insulin resistance. However, the observed differences could be due to dissimilarities in characteristics of strain/species of experimental animal and the nature of fat diet used [12,17,18,31,32]. Different breeds are not always physiologically the same. They can show different reactions based on their sensitivity to various environmental conditions. Since Sprague-Dawley and Albino Wistar rats are from diverse breeds, their colonies can present genetic variability throughout the world. The diversity of study designs could also be a contributing possible explanation [34]. It is therefore acceptable that studies conducted in different parts of the world may not demonstrate similar findings in spite of similar conditions.

The sex, age and length of exposure of the rats to high fat and/or fructose diet could also be a contributing effect. For instance, different metabolic reactions occur to similar diet at different age of rats [5]. This is consistent with the report presented by Han *et al*., [40] that high-fat diet altered the blood glucose level of young rats, hence the age and gender of the rats is of paramount significance. The choice of gender in experimental animal is of vital significance due to hormonal differences. Sex hormone effects can be contradicting in different animal model, hence it has been suggested earlier that gender should be considered in the choice of animal model of disease [8,31]. In this study, matured adult male rats were used because human T2D tend to occur later in life. This was contrary to vast majority of HFD/STZ rats reported in literature using young rats [19].From our preliminary investigations matured adult rats also tend to withstand the stress of STZ induction much better than the younger counterpart. Secondly, male gonadectomy has been reported to protect against diabetes in some models but ineffective or increases the incidence in other models [31].

Quite a number of available animal models of T2D are either based on high-fat diet or high-fructose [8,24,25,28]. According to Willett [5], high fat diet model stimulates glucose intolerance with hypercholesterolemia and impaired pancreatic function, whereas, the high fructose model induces hyperinsulinemia and hypertriglyceridemia. Both hypertriglyceridemia, hyperinsulinemia and hypercholesterolemia were present in the FDF fed groups. Kante *et al* [41] demonstrated that high carbohydrate and fat diet does not induce frank diabetes in experimental rats. The blood glucose level in both diet regimens does not get to the diabetic threshold; thus, it remains unsuitable for the screening of pharmacological compounds or validation of antidiabetic drugs, like insulin secretagogues or insulin sensitizers [9,36]. This observation by Kante *et al*[41]was similar to ours hence the need to treat the animals with a low dose of diabetogenic agent. The significantly higher total cholesterol and triglyceride levels in the FDF-fed after the 6 weeks dietary regimen could be the reason for the observed higher insulin resistance in the group compared to the NDF-fed group. Interestingly, the low diabetogenic dose that results in frank hyperglycaemia in the FDF groups failed to exhibit same effects in the NDF groups.

The rat model of T2D described in the present investigation, differed in the sense that they received both high fat and high fructose in their diet. This combination synergizes their metabolic outcomes, a procedure that renders rat mild hyperglycaemic, obese, insulin resistant, hypercholesterolemic, and hypertriglyceridemic with compensatory hyperinsulinemia. They also exhibited reduced glucose tolerance with significant differences (p<0.05) revealed during OGTT. Regrettably, no baseline exists for this parameter in Wistar rats [34]. Reduced glucose tolerance is comparable to prediabetic insulin resistant state in humans. Previously, it was reported that 7 weeks old male Sprague-Dawley rats fed 40% HFD had similar blood glucose concentrations to chow-fed rats, but significantly higher insulin, free fatty acid and triglyceride concentrations indicating insulin resistance [29]. More often than not, insulin resistance is overcome by β-cell-induced insulin overproduction; ultimately, the conversion of prediabetes to frank hyperglycaemia in patients having type 2 diabetes becomes linked with a decline in the secretory ability of the overwhelmed pancreatic β-cells. For that reason, the development of frank hyperglycaemia in this study was accomplished in insulin-resistant FDF-fed rats using a single low dose of STZ.

High-fat diet (HFD) is known to precipitate insulin resistance [5]. Insulin resistance presents hyperinsulinemia. This situation was noticed in this study between the FDF fed group and the NDF fed group. In insulin resistant state, there is a higher amount of circulating serum insulin when compared to normal control state. In a recent investigation, it was revealed that insulin resistance helps conserve the brain’s glucose supply by preventing muscles from taking up excessive glucose [42]. The observation of 117.5% circulating insulin in the FDF-fed control group in this study was consistent with that of the HFD-fed groups reported by Zhang *et al* [12] with 178.1%, and Srinivasan *et al* [24] with 178.4% residual insulin. The development of T2D model was accomplished in the insulin resistant FDF-fed rats upon injection with a relatively low dose of STZ (35 mg/kg bw.) which resulted in frank hyperglycaemia and a slight decrease in circulating serum insulin (85.8%). But with respect to the NDF-fed group, the same dose of STZ failed to produce obvious hyperglycaemia.

When high percentages of endogenous β-cells are destroyed, there will be little endogenous insulin production hence hyperglycaemia and weight loss [30]. The reduction in endogenous insulin production precipitates the onset of hyperglycaemia[43]. These attributes were observed in the present study. The onset of hyperglycaemia is believed to occur when 80–95% of the β-cells mass are lost [44]. In T1D the beta-cells are almost completely destroyed when patients are diagnosed. This remark was in agreement with our finding in the group administered 45 to 55mg/kg bw STZ in both the NDF-fed and FDF-fed groups. The level of impairment in β-cell function also contributes to the emergence of hyperglycaemia[43]. The magnitude of β-cell destruction varies with age and depends on the insulin producing capacity, which is proportional to β-cell mass and insulin demand which is proportional to body weight [43] an additional 3–5% may be lost in each subsequent year. Insulin secretory defect in T2D does not primarily result from insufficient β-cell mass rather from an impairment of insulin action [21,22]. The UK prospective diabetes study has shown that by the time persons are diagnosed with T2D, their β-cell function is about 60% of normal. Whether the 40% reduction is the result of cells secreting less insulin or decrease in β-cell mass is unclear. However, it has been reported that the anatomical presence of β-cells do not necessarily correlate to β-cell function [45], but the measurement of serum c-peptide and/or endogenous insulin can be used to assess β-cell function [30]. In this study, serum insulin and c-peptide were measured in order to assess β-cell function. The assessment of β-cell function is highly indispensable in understanding the pathways that lead to the inability of the β-cell to secrete adequate quantity of insulin. High insulin levels can indirectly indicate IR [30]. In this study the FDF-fed group exhibited insulin resistance.

The evaluation of percentage change in serum c-peptide and available circulating serum insulin would be a contribution to knowledge from this study especially for researchers and investigators in the choice of a specific animal model for diabetic type. It was observed that the FDF-fed group treated STZ (35 mg/kg bw) had a 12.2% decrease in serum c-peptide which consequently resulted in 85.8% available circulatory serum insulin and frank hyperglycaemia. This was contrary to the model of T2D presented by Suman *et al*[46] which had 45.51% insulin, Wilson and Islam [25] which had 53.7% serum insulin, but agrees with that of Zhang *et al* [12] with 83.1%, Masiello *et al* [47] with 88.2% and Srinivasan *et al* [24] with 83.0% of circulating serum insulin respectively. These model featured similarities of the human T2D. Interestingly, Dhansh *et al*., [48] reported a model of T1D developed using 45 mg/kg bw. STZ and they observed 28.2%, 21.7% and 19.8% available serum insulin, after 15, 30 and 45 days post STZ induction respectively. These values are similar to those of human T1D [19, 30]. The residual circulatory insulin levels in the rat model of diabetes reported by Suman *et al* (45.51% insulin) [46], Wilson and Islam (53.7% insulin) [25] and Dhansh *et al*., (28.2%, insulin) [48] proved that the model are for diabetes type 1 and not type 2. In this study we observed that in both the NDF and FDF-fed groups, with 45 & 55 mg/kg STZ frank hyperglycaemia was developed with < 46.8% serum-insulin, a severe deficiency typical of diabetes type-1 [19,30]. This value was synonymous to that of Suman *et al* in 2016 [46] and that of Wilson and Islam in 2012 [25]. The NDF_25_ and NDF_35_ groups with 75.7 and 64.4% serum insulin respectively presented relatively normoglycemia, whereas the FDF_35_ (85.75% serum insulin) were notably hyperglycaemia (>300 mg/dL). It is obvious from this result that the FDF_35_ group developed diabetes because they were already insulin resistant coupled with hyperinsulinemia, a condition needed to sustain normal blood glucose level. So, the insignificant low dose of STZ presenting normoglycemia in NDF-fed group resulted in compromise of the beta cell function in the FDF-fed group. In the NDF_35_ group, the effect of STZ (35mg/kg bw) could possibly have been compensated by the normal defense homeostasis mechanisms, unlike the FDF_35_ group which were already insulin resistant presenting mild hyperglycaemia.

The blood glucose levels in the FDF_35_ model were observed to be relatively stable throughout the 6weeks post diabetes validation of the study. This implies that the model could be valuable for chronic diabetes investigations like hypertension, neuropathy and nephropathy. The suitability of the model for pharmacological screening was validated using glibenclamide, metformin and pioglitazone. The ability of these drugs to reduce the total cholesterol, triglyceride and the blood glucose level justify the model for pharmacological screening. This is the first study in which three different drugs were used to validate a model.

Researchers in this part of the country and in the rest of the world would find the unique rat model of type 2 diabetes presented in this report exceptional as it addresses some key issues lacking in earlier reported model. Firstly, the diet composition described by Sasidharan *et al*., [17], Wang *et al*., [42] and Srinivasan *et al*., [24] are not readily available to researchers in this part of the country unlike the one reported here. Secondly, it is well known that the amount of circulating C-peptide can be used to ascertain the nature of the β-cell mass. These biomarkers were also not accounted for by previous investigators [12,24,25,47]. Although they measured the serum insulin level which is known to be secreted into the blood stream in equimolar ratio with C-peptide, the amount of viable β-cell mass available at the point of diagnosis is a determinant for the type and/or phase (early or late) of diabetes under investigation [49]. For instance, it has been reported that 90–95% of β-cell mass is lost at the point of diagnosis of type 1 diabetes whereas a relative amount (24–30%) is lost within 5 years of diagnosis of type 2 diabetes. In another report a 54–60% of β-cell mass is said to have been lost after 15 years of diagnosis of type 2 diabetes [50]. In this study only a relative 12.16% C-peptide was lost in the FDF_35_ model unlike >47.3% observed in the 45 and 55 STZ treated groups in NDF-fed and FDF-fed.

One limitation in this study is the unavailable means to determine the amount of available β-cell mass and to perform insulin tolerance test. However, further investigation is in progress to determine the diabetogenic dose that would results in both hyperglycaemia and normoinsulinemia in animals.

## Conclusion and Recommendation

The present study has established a benchmark of residual insulin levels in selecting animal model for T2D studies. The experimental rat model of T2D presented in this study is a classical replica of the westernized unhealthy lifestyle seen in human T2D subjects, in which fructose (20%) in drinking water is supplied together with high fat diet (Margarine fortified diet feed) to produce insulin resistance. The subsequent injection of sub-diabetogenic dose of streptozotocin (35 mg/kg bw) elevated the blood glucose to the diabetic level. In view of the above observations, it was hereafter recommended that the animal model intended for diabetes studies should be examined in order to ascertain the percentage available circulating endogenous serum insulin and c-peptide as a measure of the type of diabetes. The results from this research strongly suggests that selection of animal model for T2D studies should be based on circulating endogenous serum insulin of not less than 85.7% of normal control groups in a hyperglycaemic condition of non-fasting blood glucose ≥300mg/dL. In addition the serum C-peptide level should in relative term be insignificantly different from the normal control group. This study revealed that a combination of margarine and fructose fortified diet-fed with single low dose (≤35 mg/kg bw.) STZ-treated rat is a suitable non-genetic model for T2D studies. The model mimics the natural history and metabolic features of human T2D. In addition, it is cheap, easy to develop and useful for evaluating and screening of therapeutic compounds intended for treatment of T2D.
